# Diagnostic Uncertainty and the Epidemiology of Feline Foamy Virus in Pumas (*Puma concolor*)

**DOI:** 10.1038/s41598-020-58350-7

**Published:** 2020-01-31

**Authors:** Nicholas G. Dannemiller, Sarah Kechejian, Simona Kraberger, Kenneth Logan, Mathew Alldredge, Kevin R. Crooks, Sue VandeWoude, Scott Carver

**Affiliations:** 1 Department of Microbiology, Immunology, and Pathology, Colorado State University, Fort Collins, Colorado USA; 2 Colorado Parks and Wildlife, Montrose, Colorado USA; 3 Colorado Parks and Wildlife, Fort Collins, Colorado USA; 4 Department of Fish, Wildlife, and Conservation Biology, Colorado State University, Fort Collins, Colorado USA; 5 School of Biological Sciences, University of Tasmania, Hobart, Tasmania Australia

**Keywords:** Ecological epidemiology, Viral infection, Risk factors

## Abstract

Feline foamy virus (FFV) is a contact-dependent retrovirus forming chronic, largely apathogenic, infections in domestic and wild felid populations worldwide. Given there is no current ‘gold standard’ diagnostic test for FFV, efforts to elucidate the ecology and epidemiology of the virus may be complicated by unknown sensitivity and specificity of diagnostic tests. Using Bayesian Latent Class Analysis, we estimated the sensitivity and specificity of the only two FFV diagnostic tests available—ELISA and qPCR—as well as the prevalence of FFV in a large cohort of pumas from Colorado. We evaluated the diagnostic agreement of ELISA and qPCR, and whether differences in their diagnostic accuracy impacted risk factor analyses for FFV infection. Our results suggest ELISA and qPCR did not have strong diagnostic agreement, despite FFV causing a persistent infection. While both tests had similar sensitivity, ELISA had higher specificity. ELISA, but not qPCR, identified age to be a significant risk factor, whereas neither qPCR nor ELISA identified sex to be a risk factor. This suggests FFV transmission in pumas may primarily be via non-antagonistic, social interactions between adult conspecifics. Our study highlights that combined use of qPCR and ELISA for FFV may enhance estimates of the true prevalence of FFV and epidemiological inferences.

## Introduction

Feline foamy virus (FFV) is a contact-dependent retrovirus that results in persistent and apparently apathogenic infections in domestic and wild cats globally^1,2^. Epidemiological investigations of FFV to date have been predominantly based on domestic cats, with few studies documenting the prevalence of FFV in wild felids^3–6^. FFV in domestic and wild cats worldwide is highly prevalent and seroprevalences vary from 8–80% based on geographic location^3–14^. Furthermore, we have documented high FFV seroprevalences in pumas *(Puma concolor)*, one of the largest and most charismatic wild felids in the Americas^15^. FFV transmission occurs through salivary shedding, with biting and amicable social contact such as grooming between cats acting as possible transmission routes^12,16,17^. Older cats have a higher prevalence of FFV, given the cumulative risk of infection as the cats age^12,16^. Sex has been suggested not to be a risk factor for FFV infection^6,12^, but male domestic cats in Colorado have recently been reported to be at higher risk for infection^18^.

Diagnostic test accuracy is typically estimated by comparing test results from a novel assay to the results of a test considered the most diagnostically accurate or where the misclassification error is reliably known (i.e., a ‘gold standard’ test). Several unique challenges exist in the development of well-characterized diagnostic tests for infections of wildlife. These challenges include: field conditions that result in collected samples being less than optimally stored or processed, the current ‘gold standard’ test cannot be performed antemortem, or no known true positive and true negative samples are available. Consequently, these limitations can result in diagnostic uncertainty that yields biased estimates of true disease prevalence^19^.

Statistical models have been developed to overcome shortcomings in defining diagnostic test accuracy. In particular, latent class analysis (LCA) has become increasingly used for improving disease prevalence estimates from imperfectly calibrated diagnostic tests^20^. LCA describes a probabilistic model relating the outcomes of one or more binary, imperfect diagnostic tests to the unknown, unobserved (“latent”) disease status. The likelihood of the LCA model is then maximized to provide estimates of the respective sensitivity and specificity for each diagnostic test included in the model as well as the overall disease prevalence. Application of LCA methodology has become increasingly common in veterinary epidemiology and has often been coupled with Bayesian and hierarchical modelling^21–25^.

While many veterinarians rarely screen felids for FFV due to its apathogenic nature, the virus’s cosmopolitan prevalence and persistent infection makes it a ‘model’ infectious agent for wildlife veterinarians and researchers studying landscape genetics or modeling the transmission dynamics of more virulent pathogens among wild felids. Thus, an accurate understanding of the ecology and epidemiology of FFV would be foundational knowledge for future research. This understanding, however, is intrinsically reliant upon the diagnostic accuracy of the tests used to determine FFV status. Our study’s objective was to compare the results of the only two FFV diagnostic tests available—qPCR and ELISA—from pumas tested by both assays to determine the diagnostic agreement between the two tests and to use Bayesian LCA to determine the sensitivity and specificity of each test as well as the prevalence of FFV. We tested pumas for FFV exposure by both quantitative PCR (qPCR), which detects and quantifies FFV viral genomes in DNA extracts of blood, and an enzyme-linked immunosorbent assay (ELISA), which detects FFV antibodies in serum or plasma. Since foamy viruses are generally considered to induce lifelong infection without elimination of viral genomes, pumas would presumably test positive for both assays following exposure, viral replication, and seroconversion. Risk factors for infection, specifically puma sex and age, were also analyzed and compared for qPCR or ELISA to determine if the epidemiological inferences differed between the two diagnostic tests.

## Methods

### Puma sampling

From 2005–2014, blood samples and demographic information (sex and age) were collected as previously described from pumas in two locations in Colorado: the Western Slope and the Front Range^26^. One hundred pumas on the Western Slope and 71 pumas on the Front Range were sampled, yielding a sample population of 171 animals for this study. All pumas were sampled in accordance with relevant guidelines and after appropriate Institutional Animal Care and Use Committee approvals were obtained from Colorado State University and Colorado Parks and Wildlife. qPCR testing targeting the FFV *gag* gene was performed using DNA isolated from either whole blood or clot as described^27–29^. Capture ELISA was performed on sera to detect anti-FFV Gag using serum or plasma as previously described^15,18,29^. Sera were tested at a 1:50 dilution, with a positive result being defined as an absorbance of two times the average of the duplicate wells of the negative control plus three times the standard deviation.

Both qPCR and ELISA were run with known FFV positive and negative samples validated by multiple laboratory personal using standardized protocols to ensure the rigor of our results. The qPCR and ELISA compared in this study were chosen due to their accepted use in the FFV literature^15,18,28–31^ and the unique opportunity that a large cohort of pumas were tested for FFV by both assays. Owing to the apparently benign nature of FFV there are no commercial FFV assays available at this time. Currently there are also no other research laboratories performing FFV assays for pumas. Nonetheless qPCR and ELISA are the standard viral diagnostics for FFV (and many other feline viruses), as evidenced by previous literature, and have been used to provide population-level surveillance of FFV infection^15,18,28–31^. The qPCR and ELISA datasets analysed during the current study are available from the corresponding author on reasonable request.

### qPCR and ELISA diagnostic agreement

Diagnostic agreement between qPCR and ELISA was evaluated using Cohen’s kappa statistic, a correlation coefficient indicating the proportion of agreement beyond that expected by chance^32^. Given that low or high disease prevalence, as well as bias, can affect kappa, a prevalence-adjusted bias-adjusted kappa (PABAK) was also calculated^33^. A McNemar’s test was used to further detect if any bias present was significant^34^. A bias index equal to the difference in FFV positive (FFV+) results between qPCR and ELISA, and a prevalence index equal to the difference between the probability of a puma being FFV+ and FFV negative (FFV−) were also calculated to aid in the interpretation of reported kappa values^33^. Statistics were calculated using the ‘epiR’ package^35^ in the free software program R version 3.4.2 (R Core Team, Vienna, Austria).

### Latent class analysis

qPCR and ELISA sensitivity (Se) and specificity (Sp) as well as FFV prevalence were estimated using Bayesian LCA methods and code adopted from Lewis and Torgerson (2012)^36^. Given Se, Sp, and prevalence can range from 0–1, a beta prior distribution was used to model parameter uncertainty^37^. Both non-informative and informative prior distributions were run. Our non-informative priors used a beta (1, 1) distribution, assuming the true value of all three parameters was between 0–1. Our informative priors made the following assumptions: qPCR Se is >0.70 with a mode of 0.75 and an 80% certainty, leading to a beta (52.65, 18.22) distribution; qPCR Sp is >0.95 with a mode of 0.99 and an 80% certainty, leading to a beta (42.99, 1.42) distribution; ELISA Se is >0.90 with a mode of 0.95 and an 80% certainty, leading to a beta (40.58, 3.08) distribution; ELISA Sp is >0.95 with a mode of 0.99 and an 80% certainty, leading to a beta (42.99, 1.42) distribution; FFV prevalence is >0.50 with a mode of 0.60 and an 80% certainty, leading to a beta (12.23, 8.48) distribution. Given FFV in domestic cats is 95–100% genetically similar to FFV in pumas on a full genome level^28,38^ and the current lack of sensitivity/specificity data for either test in pumas, informative priors were based on authors’ expert experience using these assays for domestic cat FFV infections^29^. Priors for Se and Sp were also informed by values from FFV experimental inoculations in domestic cats in^29^ (see also Supplementary Table 1). Beta distributions were calculated using the ‘epiR’ package in R. To account for possible conditional dependence between PCR and ELISA, an LCA model with covariance between Se and Sp of the two tests were run with both sets of priors. LCA models were fitted using Markov chain Monte Carlo (MCMC) estimation using the free software JAGS (Just Another Gibbs Sampler) version 4.3.0^39^ and the ‘coda’ package^40^ in R. Our MCMC estimation ran three independent chains for 20,000 iterations, after a burn-in of 10,000 iterations and thinning of 10, yielding 1,000 values to derive parameter posterior means. Gelman-Rubin statistics^41^ were used to assess convergence through the ‘coda’ package.

### Risk factor analysis

Sex (male/female) and age (young/adult) were evaluated as potential risk factors for FFV infection, as determined by qPCR and ELISA, using Bayesian Generalized Linear Mixed Models (GLMMs) with sex and age as fixed effects and location as a random effect to control for potential sampling bias. Pumas with an unknown or unrecorded sex and age were excluded from risk factor analyses. Coefficients were estimated using MCMC through the ‘MCMCglmm’ package^42^ in R. Models ran for 50,000 iterations, after a burn-in of 10,000 iterations and thinning of 10, yielding 4,000 values to derive parameter posterior means. Convergence was assessed visually by comparing the posterior distributions of multiple runs of each model. LCA and GLMM models were compared using the Deviance Information Criterion (DIC); models with lower DIC values were better supported by our data and subsequently had greater model fit. Models within 2 DIC units were considered indistinguishable. Model weights and averaged coefficients were also calculated to examine the effect sizes of variables.

## Results

Due to variation in sample quantity, quality, and availability, 171 pumas were tested for FFV with qPCR and 128 were concurrently tested with ELISA. Overall, qPCR and ELISA showed a 75.8% (97/128) agreement and a 24.2% (31/128) disagreement in pumas tested for FFV. Twenty-four pumas (18.8%) tested FFV− and 73 (57.0%) tested FFV+ by both qPCR and ELISA. Twenty-six pumas (20.3%) were seropositive but qPCR negative and five pumas (3.9%) were qPCR positive but seronegative. The kappa statistic for qPCR and ELISA was 0.45, with a bias index of 0.16 and a prevalence index of −0.38. The PABAK statistic was 0.52, showing that mathematical adjustments accounting for bias and prevalence improved the reported agreement between qPCR and ELISA. A McNemar’s test comparing qPCR and ELISA yielded a test statistic of *X*^2^ (1, *N = *128) = 14.23, p-value < 0.001 further indicating there was significant bias between the two diagnostic tests.

**Figure 1:**
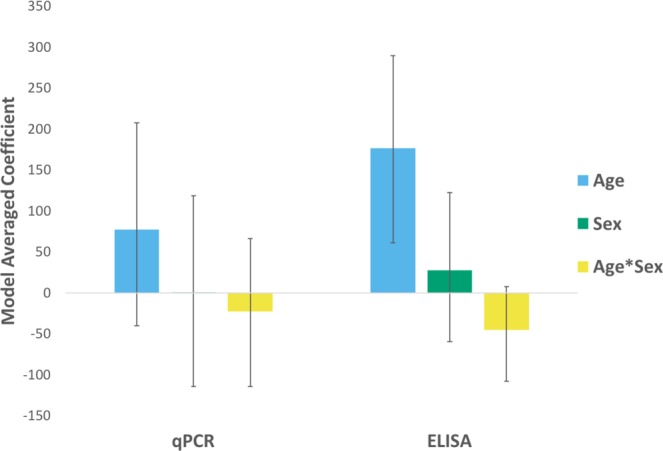
FFV ELISA and qPCR differ in predictions of age as a risk factor for infection. Average model coefficients of risk factors for FFV infection as determined by qPCR and ELISA. Although qPCR and ELISA both found sex not to be a significant risk factor, ELISA (but not qPCR) found age to a significant risk factor.

Gelman-Rubin statistics indicated all Bayesian LCA models adequately converged, with each parameter’s upper 95% confidence limit approximately equal to 1. Our LCA model with uninformative priors and covariance between qPCR and ELISA was deemed the most parsimonious model (Table 1). Weighted averages of the LCA models found qPCR and ELISA had similar Se, whereas ELISA had a greater Sp than qPCR (Table 1). The estimated true FFV prevalence (94.8%) was substantially greater than the apparent FFV prevalence determined by either qPCR (60.9%) or ELISA (77.3%). Plots of posterior distributions of multiple runs indicated that GLMMs adequately converged. For both qPCR and ELISA, model weights indicated that the predictor variables age, sex, and the interaction between age and sex provide the most parsimonious model (Table 2). Neither sex nor age were significant risk factors for FFV infection as determined by qPCR; however, age (but not sex) was significant risk factor for FFV infection as determined by ELISA (Fig. 1).

**Table 1:** Latent Class Analysis estimates true prevalence of FFV to be 94.8%.

Model	qPCR		ELISA		Prevalence (%)	DIC	ΔDIC	*w*
	Se (%)	Sp (%)	Se (%)	Sp (%)				
UIP & Cov	66.1 ± 4.2	42.3 ± 21.4	65.4 ± 4.3	45.9 ± 21.8	94.8 ± 5	241.2	0	1.0
IP & Cov	74.2 ± 3.3	94.6 ± 3.5	80.2 ± 5.1	95.0 ± 3.2	85.0 ± 4.5	261.2	20	0.0
UIP	83.0 ± 8.5	87.5 ± 8.4	95.3 ± 3.3	67.9 ± 16.5	69.0 ± 8.8	272.9	31.7	0.0
IP	74.5 ± 3.4	96.5 ± 2.7	94.3 ± 2.2	96.3 ± 3	77.6 ± 3.8	282.2	41	0.0
Model Average	66.1 ± 4.2	42.3 ± 21.4	65.4 ± 4.3	45.9 ± 21.8	94.8 ± 5			

Average model coefficients ± standard deviation for the sensitivity (Se) and specificity (Sp) of qPCR and ELISA as well as true prevalence of FFV based on Bayesian Latent Class Analysis. Deviance Information Criterion (DIC) values and model weights (*w*) suggest uninformative priors and covariance between qPCR and ELISA to be the most parsimonious model. UIP = uninformative priors; IP = informative priors; Cov = covariance.

**Table 2:** Age, sex, and their interaction best predict FFV infection.

qPCR Model	DIC	ΔDIC	*w*	ELISA Model	DIC	ΔDIC	*w*
Age + Sex + Age*Sex	3.0	0.0	0.38	Age + Sex + Age*Sex	1.6	0.0	0.27
Age + Sex	3.7	0.7	0.27	Age + Sex	1.7	0.1	0.26
Age	4.1	1.1	0.22	Age	2.1	0.5	0.21
Sex	5.1	2.1	0.13	Sex	2.2	0.6	0.20
Null	29.5	26.5	0.00	Null	4.3	2.7	0.07

Deviance Information Criterion (DIC) values and model weights (*w*) suggest the predictor variables age, sex, and the interaction between age and sex provide the most parsimonious model for FFV infection for both qPCR and ELISA. Age*Sex = the interaction between age and sex.

## Discussion

While the ecology and epidemiology of a putatively apathogenic virus in wildlife may seem moot, FFV and other pathogens that cause persistent, apathogenic infections are ‘model’ infectious agents for landscape genetics because infected individuals at any time post-infection and continue normal behaviors^43^. Consequently, the accuracy of currently employed FFV diagnostic tests has downstream impacts on the accuracy of FFV landscape genetics exploring wild felid social structure and movement patterns. as well as the potential transmission dynamics of pathogens with higher virulence such as feline leukemia virus, a major threat to the critically endangered Florida panther (*Puma concolor coryi*)^44^. To aid future landscape genetic studies, we estimated the Se and Sp of two common FFV diagnostic tests—qPCR and ELISA—within a large sample of Colorado pumas. For our study, Se is the ability to correctly identify FFV+ pumas and Sp is the ability to correctly identify FFV− pumas.

Our results suggest ELISA and qPCR had similar estimated Se, whereas ELISA had a higher estimated Sp than qPCR implying that ELISA is a stronger confirmatory test for assessment of FFV infection. It should be noted that the Se and Sp estimates reported here are currently the only ELISA and qPCR assays available for FFV, not currently being used by other labs and thus in-house. It is possible that the Se and Sp of the ELISA and qPCR used in our study could vary if performed by other laboratories, researchers, or in a different cohort of pumas. To minimize this potential limitation in reproducibility both our qPCR and ELISA were run with known FFV positive and negative samples using the standardized protocol validated by multiple laboratory personal to ensure rigor of results. Nevertheless, future comparative studies evaluating the diagnostic agreement between different laboratories or personnel running the same FFV assay on mutual samples may be valuable for both epidemiological investigation and diagnostic purposes.

Although pumas could be qPCR positive while seronegative during early infection or if antibody titers are below the limit of ELISA detection, it is more likely that low levels of FFV replication and antigenemia would cause antibody titers to persist, even during the quiescent phase of viral infection when FFV proviral load falls below the limit of qPCR detection or if viral genetic mutations occurred in the qPCR primer or probe binding sites. This finding is important, as it indicates to researchers and wildlife managers that a seropositive puma is more likely to be truly FFV+ and should be included in subsequent landscape genetic modeling efforts. However, neither qPCR and ELISA’s estimated Se or Sp approached 100%, indicating diagnostic uncertainty in either test’s results and offering an explanation for why the estimated true prevalence of FFV was higher than the apparent FFV prevalence determined by either method. Our analysis reinforces that reliance on a single imperfect diagnostic test likely underestimates disease prevalence. Future research could seek to confirm early FFV infection in qPCR-positive, ELISA-negative pumas by looking for anti-FFV IgM to confirm the qPCR as a true positive as well as quiescent FFV infection in qPCR-negative, ELISA-positive pumas by quantifying FFV RNA to confirm the ELISA was a true positive. However, recent work suggests plasma RNA is often below limits of detection and therefore less sensitive for detection of infection than PBMC proviral load^31^ and that FFV RNA is more likely to be isolated from saliva than blood^45^ making the confirmation of qPCR-negative, ELISA-positive pumas potentially difficult. Overall, research may benefit from simultaneous use of both diagnostics.

Our low kappa statistic implies there is diagnostic disagreement between qPCR and ELISA, a finding supported by the associated bias and prevalence indexes and PABAK. Given our prevalence index is larger than our bias index and our PABAK is greater than our kappa statistic, prevalence has a greater effect on our kappa statistic than bias. Prevalence in this context is the probability of a puma being FFV+, which is high given the widespread occurrence of FFV infection in this cohort of pumas. Consequently, a high prevalence means the potential chance agreement between qPCR and ELISA is also high and our kappa statistic is reduced accordingly. The bias affecting our kappa statistic and detected by the McNemar’s test is the difference in FFV+ results between qPCR and ELISA. This bias is significant due to the large number of pumas determined to be FFV+ by ELISA, but FFV− by qPCR. This discordance could be due to infected pumas remaining seropositive, despite having circulating proviral loads that fall below the limit of qPCR detection or clearing the infection^29^. Fewer pumas were determined FFV+ by qPCR and FFV− by ELISA, a discrepancy more reflective of acute infections that were measured before the development of detectable antibodies.

Given their Se and Sp covariance, our models suggest that qPCR and ELISA are not conditionally independent and neither test provides independent evidence of the presence/absence of FFV infection. This is plausible because although qPCR and ELISA determine FFV infection via different mechanisms (DNA vs. antibody), persistently FFV− infected pumas can be seropositive and provirus-positive simultaneously, making a qPCR FFV+ result not mutually exclusive to an ELISA FFV+ result. A larger Se than Sp covariance suggests qPCR and ELISA would have a greater reduction in Se when interpreting the two tests in parallel, but less of a reduction in Sp when interpreting the two tests in series^46^. Consequently, researchers and wildlife managers could have greater confidence in classifying a puma as truly FFV+ by running ELISA after qPCR identifies the puma as FFV+ or vice versa.

Neither qPCR or ELISA associated sex as a significant risk factor for FFV infection, supporting previous studies of FFV in domestic cats^6,12^ and pumas in other states^15^, but contrasting recent FFV studies in domestic cats in Colorado^18^. This difference in risk factors within Colorado suggests an underlying difference in the risk of FFV exposure in pumas and domestic cats. Interestingly, a similar pattern has been noted between domestic cats infected with feline gammaherpesvirus (GHV), with male cats being at greater risk for infection^47^, versus GHV infections in bobcats or pumas, which show males and females of these nondomestic species at equal risk for infection^47,48^. It is possible that FFV transmission in pumas is primarily via social contact between familial groups, whereas transmission in feral domestic cats is more likely to occur via male cat interactions. Although regarded as solitary apex predators, pumas can frequently interact with adult conspecifics, supporting the possibility of pathogen transmission through social contact^49^. It is also possible there is a biological susceptibility of male domestic cats to these infections or an unidentified aspect of their life history that places them at greater risk for infection.

ELISA, but not qPCR, found age to be a significant risk factor for FFV infection in pumas with adults being more likely to be seropositive due to the cumulative risk of infection over a puma’s lifetime. Unsurprisingly, this risk association was found with ELISA which is better able to detect historical FFV infection compared to qPCR. Given the estimated differences in Sp between qPCR and ELISA, we believe ELISA to have fewer false positives and subsequently the ELISA GLMMs to be more accurate. Age has been previously noted to be a risk factor for FFV infection, suggesting exposure occurs during conspecific interactions that occur after birth and weaning^12,16^. This epidemiological discrepancy in age as a risk factor for FFV infection underscores that diagnostic uncertainty can confound our ability to infer the transmission dynamics of wildlife diseases. Our work highlights the need to select the appropriate diagnostic test based upon the objective of the study being conducted. ELISA has a higher Sp, making it more accurate for descriptive epidemiological studies and risk factor analyses, whereas qPCR allows estimates of proviral load, which may be more relevant for determining infection kinetics, viral replication rates, or transmission dynamics.

Our estimated true FFV prevalence in Colorado pumas is markedly higher than the range of FFV prevalences found in other wild felid species^3–6^. This variance in prevalence between host species could potentially be due to differences in sample size or the unaccounted diagnostic uncertainty of the FFV tests employed by previous studies. This analysis illustrates the importance of understanding diagnostic uncertainty when interpreting disease parameter estimates and making epidemiological inferences. Given the broad and established literature on estimating diagnostic accuracy and disease prevalence in the absence of a ‘gold standard’ diagnostic test, disease ecologists and wildlife epidemiologists should continue to use flexible and robust statistical frameworks when using imperfect diagnostic tests to study pathogens in wild populations.

## Supplementary information

Supplementary Table S1.
