# Supplementary material for: Diagnostic Uncertainty and the Epidemiology of Feline Foamy Virus in Pumas (Puma concolor)
Source: Sci Rep. 2020 Jan 31;10:1587. doi: 10.1038/s41598-020-58350-7 (PMC6994588; doi:10.1038/s41598-020-58350-7)
Supplement: Supplementary file 1 — Supplementary Table S1. [file 41598_2020_58350_MOESM1_ESM.pdf]

Diagnostic Uncertainty and the Epidemiology of Feline Foamy Virus in Pumas (*Puma concolor*)

Nicholas G. Dannemiller[*a], Sarah Kechejian[a], Simona Kraberger[a], Kenneth Logan[b], Mathew Alldredge[c], Kevin R. Crooks[d], Sue VandeWoude[a], Scott Carver[e]

[a]Department of Microbiology, Immunology, and Pathology, Colorado State University, Fort Collins, Colorado, USA

[b]Colorado Parks and Wildlife, Montrose, Colorado, USA

[c]Colorado Parks and Wildlife, Fort Collins, Colorado, USA

[d]Department of Fish, Wildlife, and Conservation Biology, Colorado State University, Fort Collins, Colorado, USA

[e]School of Biological Sciences, University of Tasmania, Hobart, Tasmania, Australia

*Address correspondence to Nicholas G. Dannemiller, dannemillern@gmail.com

Supplementary Table S1. Results of qPCR and ELISA testing of naïve and experimentally FFV-inoculated domestic cats. Four specific-pathogen-free domestic cats were experimentally inoculated with 10^5 $TCID_{50}$ wild-type FFV in 2 ml, split into 1 ml intramuscularly and 1 ml intravenously. qPCR and ELISA testing of the 4 experimentally FFV-inoculated cats as well as 4 additional specific-pathogen-free, FFV-naïve domestic cats 21-days post inoculation yielded a sensitivity of 75% (95% CI: 19.4-99.4%) and specificity of 100% (95% CI: 39.8-100%) for both qPCR and ELISA. Data extrapolated from Ledesma-Feliciano et al (2018)[28].

Cat	qPCR+	qPCR-	ELISA+	ELISA-
FFV+	3	1	3	1
FFV Naïve	0	4	0	4
